# Objective Measurement of Physical Activity and Sedentary Behavior in Patients with Chronic Obstructive Pulmonary Disease: Points to Keep in Mind during Evaluations

**DOI:** 10.3390/jcm12093254

**Published:** 2023-05-02

**Authors:** Yoshiaki Minakata, Yuichiro Azuma, Seigo Sasaki, Yusuke Murakami

**Affiliations:** National Hospital Organization Wakayama Hospital, 1138 Wada, Mihama-Cho, Hidaka-gun, Wakayama 644-0044, Japan; azuma19841025@yahoo.co.jp (Y.A.); sasaki.seigo.su@mail.hosp.go.jp (S.S.); murakami.yusuke.am@mail.hosp.go.jp (Y.M.)

**Keywords:** accelerometer, indicator, environmental factor, methodology, reproducibility

## Abstract

Objective measurement methods using accelerometers have become the mainstream approach for evaluating physical activity (PA) and sedentary behavior (SB). However, several problems face the objective evaluation of PA and SB in patients with chronic obstructive pulmonary disease (COPD). For example, indicators of PA differ depending on whether the accelerometer detects the kind of activity on the one hand, or its intensity on the other. Measured data are also strongly influenced by environmental factors (weather, season, employment status, etc.) and methodological factors (days with uncommon activities, non-wearing time, minimum required wearing time per day, minimum number of valid days required, etc.). Therefore, adjusting for these factors is required when evaluating PA or SB, especially when evaluating the effects of intervention. The exclusion of sleeping time, unification of total measurement time, and minimization of the required wearing time per day might be more important for the evaluation of ST than for evaluating PA. The lying-down-time-to-sitting-time ratio was shown to be larger in COPD patients than in healthy subjects. In this review, we clarified the problems encountered during objective evaluations of PA and SB in patients with COPD and encouraged investigators to recognize the presence of these problems and the importance of adjusting for them.

## 1. Introduction

**Figure 1:**
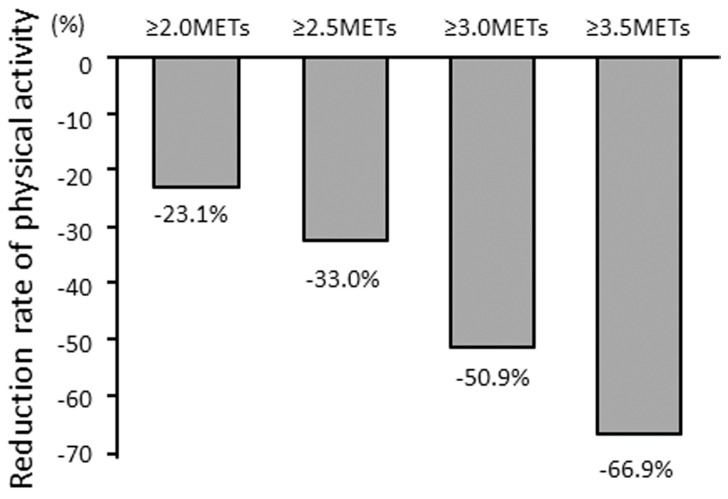
Mean reduction rate of PA in COPD patients compared to healthy subjects. Mean reduction rate of PA in COPD patients was calculated as 100 × [(mean duration of PA in COPD) − (mean duration of PA in healthy subjects)]/(mean duration of PA in healthy subjects) at each intensity of PA. PA: physical activity; COPD: Chronic obstructive pulmonary disease; METs: metabolic equivalents. Quoted from reference [5].

Chronic obstructive pulmonary disease (COPD) is now a major cause of morbidity and mortality worldwide [1,2], and its burden is projected to increase in the coming decades because of continued exposure to COPD risk factors and the aging of the world’s population [3]. Physical activity (PA) is lower in COPD patients than in healthy subjects [4,5] (Figure 1). A reduced level of PA was associated with a decline in forced expiratory volume in one second (FEV1) [6,7,8,9,10], COPD exacerbation [11,12,13,14,15], and mortality [11,16,17]. Furthermore, lower PA has been shown to be the strongest predictor of all-cause mortality in patients with COPD [17].

In COPD, hyperinflation causes exertional dyspnea, leading to a vicious cycle of a reduced exercise capacity, decreased PA, skeletal muscle dysfunction, and further dyspnea, thereby equating to a poor prognosis [18]. Exercise capacity reflects a patient’s maximal ability to do exercise, while PA reflects a patient’s willingness to move. PA is defined as any bodily movement by skeletal muscle that results in energy expenditure [19] but is usually taken to mean physically active behavior that is comparable to moderate-to-vigorous-intensity PA (MVPA) [20,21].

The two parameters of “physical inactivity” and “sedentary behavior (SB)” are also considered when evaluating a subject’s physical condition. Physical inactivity is defined as a PA level that is not sufficient for meeting present PA recommendations, which is 150 min of MVPA per week or 75 min of vigorous-intensity PA per week, or an equivalent combination of moderate- and vigorous-intensity activity [21,22]. Physical inactivity is simply the opposite of PA. SB is defined as any waking behavior characterized by an energy expenditure ≤1.5 metabolic equivalents (METs) while in a sitting, reclining, or lying-down posture [21,23,24]. As SB is a risk factor for COPD mortality independently of PA [25], it has been attracting an increasing amount of attention in recent years [25,26,27,28,29,30,31].

Objective measurement methods using accelerometers have become mainstream approaches for evaluating PA and SB, replacing conventional questionnaire-based methods, which tended to overestimate the findings [32]. However, objective approaches still involve several issues that remain to be resolved. While data obtained by objective measurements are thought to be highly accurate, no adjusting for influencing factors can reduce the reliability and significantly affect results, especially in intervention studies. Furthermore, PA is behavior with a relatively high intensity and accounts for only a small part of the day, whereas SB has a low intensity and accounts for most of our waking hours. This should be kept in mind when evaluating PA or SB with an accelerometer.

In this narrative review, we clarified the problems encountered during objective evaluations for PA and SB in patients with COPD and encouraged investigators to recognize the presence of these problems and the importance of adjusting for them.

## 2. The Objective Measurement of PA in COPD

### 2.1. Self-Reported vs. Objectively Measured PA

**Figure 2:**
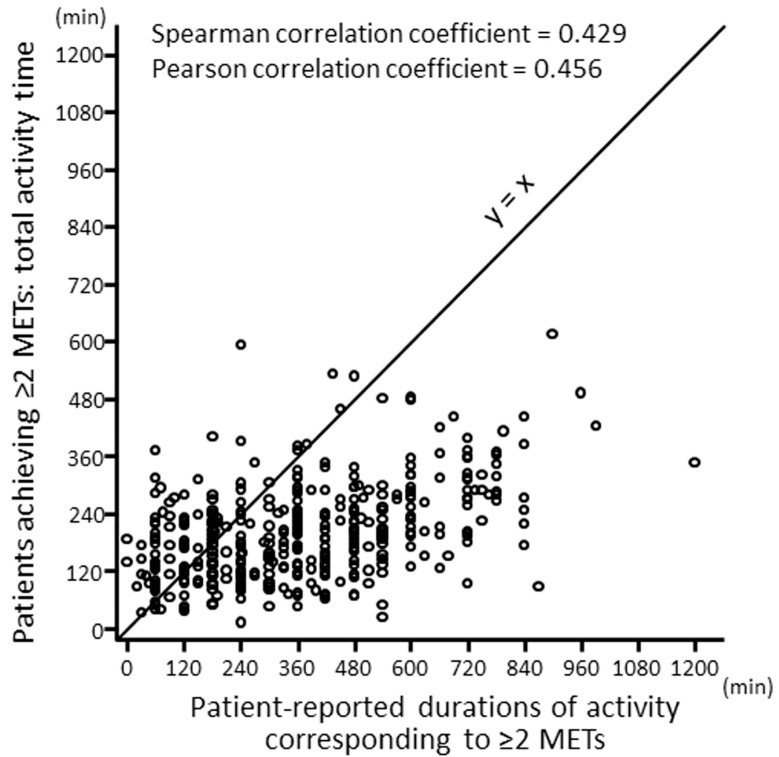
Correlations between objectively measured and patient-reported durations of PA equivalent to ≥2 METs. PA: physical activity; METs: metabolic equivalents. Quoted from reference [32] with permission.

When the duration at ≥2.0 METs measured by a questionnaire and that by an accelerometer were compared in COPD patients, most of the patients showed higher values based on a questionnaire than based on an accelerometer evaluation (Figure 2), and the mean (±standard deviation [SD]) values were 366.7 (221.8) min and 201.2 (99.1) min, respectively [32]. When the duration at >3.0 METs measured by a questionnaire and that by an accelerometer were compared, these values were 146 (143.1) min and 65 (89.4) min, respectively [33]. A systematic review showed that self-reported assessments overestimate the level of PA compared with objectively measured assessments [34]. Therefore, the objectively measured assessments are accurate, although they have various technical and associated difficulties.

### 2.2. Types of Accelerometry

Accelerometers are roughly classified into two types: those that detect the kinds of activity undertaken (e.g., DynaPort MoveMonitor™ from McRobert BV, the Hague, The Netherlands) and those that detect the intensity of activity (e.g., SenseWear Armband™ from BodyMedia Inc., Pittsburgh, PA, USA; Active Style Pro HJA-750C™ from Omron Health Care, Kyoto, Japan; etc.). Both types of accelerometers can measure total PA and the daily step count. In the type that detects intensity of activity, the results are expressed as acceleration for some models and activity intensity for others.

### 2.3. Indicators

There are various indicators for assessing PA, depending on the type of accelerometer used. When accelerometers that detect kinds of activity are used, the duration of walking and/or standing, proportion of the duration of walking and/or standing to that of total activity, movement intensity during movement, and total step count can be used as indicators. When accelerometers that detect intensity of activity are used, durations of several intensities, e.g., light PA (LPA; 1.5–3.0 METs), moderate PA (MPA; 3.0–6.0 METs), vigorous PA (VPA; ≥6.0 METs), moderate-to-vigorous PA (MVPA; ≥3.0 METs), LPA + MVPA (≥2.0 METs), total PA at ≥3.0 METs (METs∙h), and step count can be used as indicators. Some accelerometers display the results in acceleration intensity rather than activity intensity. The values for these metrics are evaluated daily or weekly.

### 2.4. Validated Accelerometers for COPD

Regarding accelerometers that detect the kind of activity undertaken, the DynaPort Activity Monitor™ (McRoberts BV) [4], which is the old version of the DynaPort MoveMonitor™, and the new DynaPort MoveMonitor™ [35,36,37] are validated for use in COPD patients. Regarding accelerometers that detect the intensity of activity, the SenseWear Armband™ (Bodymedia Inc.) [34,35,37,38], RT3™ (StayHealthy Inc., Monrovia, CA, USA) [35,37,39], Actiwatch Spectrum™ (Philips Respironics, Bend, OR, USA) [35,37], Actigraph GT3X™ (Actigraph LLC, Pensacola, FL, USA) [37,40], Lifecorder™ (Kenz Suzuken Co. Ltd., Nagoya, Japan) [35,37], Actimarker™ (Panasonic, Osaka, Japan) [41], and Active Style Pro HJA-750C™ [42] have been validated for use in COPD patients.

While pedometers tended to detect low step counts for slow-walking people such as those with COPD [43], triaxial accelerometers including the DynaPort™ [44], SenseWear Armband™ [45], and Actigraph GT3X™ [46] have been able to detect slow walking. Furthermore, the Active Style Pro HJA-750C™ uses different algorithms for two different kinds of activities (household and locomotive activities), so it may be more useful for monitoring COPD patients, who often engage in low-intensity activity [47,48].

### 2.5. Environmental Factors Requiring Adjustments for Evaluations

Environmental factors can influence the PA level. These factors should be included in the individual’s average PA level, but they can also influence the results when comparing changes in PA over time. As these factors can lead to intra-patient errors, they should be minimized for longitudinal evaluations.

#### 2.5.1. Weather

Weather is one such environmental factor. The duration of PA and step count are significantly reduced on rainy days in comparison to non-rainy days [41,42,49,50,51]. Indeed, the duration of PA at ≥3.0 METs was shown to be 11.1 min on rainy days and 21.3 min on non-rainy days [41]. Furthermore, the daily step count was 3999 on non-rainy days and 3771 on rainy days [49], although this difference was below the minimal clinically important difference. Rainfall of 10 mm translated to a decrease of approximately 175 steps [50].

#### 2.5.2. Season

Season is another potential environmental factor, as the duration of PA is longer in summer than in winter [52,53,54]. Temperature might be the main factor associated with these seasonal effects. For example, when the average temperature was ≤20.5 °C, more COPD patients went out as the temperatures became warmer (odds ratio [OR]: 1.028 per 1 °C rise in temperature), and at <2.5 °C, the increase in patients going outdoors with rising temperature grew significantly (OR: 1.13 per 1 °C rise). However, when the temperature was >20.5 °C, patients reduced outdoor activity (OR: 0.96 per 1 °C rise) [55]. When the average temperature was ≤22.5 °C, the daily step count increased 43 steps per 1 °C rise, and at >22.5 °C, the daily step count fell by 891 steps per 1 °C increase in temperature [49]. The daily step count increased 316 steps for each 10 °C rise in temperature [50]. The duration of daylight time may also influence PA [52].

#### 2.5.3. Day of the Week

The day of the week might also influence PA. The PA on weekends was shown to be reduced compared to weekdays in healthy subjects [56,57,58]. However, the PA on weekends was not significantly different from that on weekdays in COPD patients [39,42]. Most healthy subjects were working, while most COPD patients were retired. The level of PA in patients with a job is higher than in those without a job [32]. Therefore, when PA is investigated in retired COPD patients, the timing of weekends or holidays might not need to be taken into consideration.

#### 2.5.4. Air Pollution

Air pollution might influence PA. In one report, the time spent outdoors decreased with increasing ozone levels but not with PM10 values. An increased ozone level decreased both the time spent outdoors and daily step count [49]. In another report, however, PA was not correlated with the values of main atmospheric pollutants, including PM10, ozone, nitrogen dioxide, and sulfur dioxide [51]. The effects of air pollution on PA are therefore still controversial.

#### 2.5.5. Employment Status

Employment status can also influence PA. The duration at ≥3.0 METs and step count in non-employed patients were significantly lower than in employed patients according to a multivariate analysis (−13.2 ± 2.9 min and −1332.3 ± 295.6 steps, respectively, compared to employed patients) [32]. Most COPD patients seem to be retired, but caution should be practiced when evaluating subjects who have a job.

### 2.6. Methodological Factors Requiring Adjustments for Evaluations

#### 2.6.1. Days with Uncommon Activities

In our daily lives, there are days when we engage in relatively uncommon activities, such as traveling or recuperating from sickness. Data from days spent engaged in these uncommon activities are therefore not representative of the usual PA and should be excluded from the analysis.

#### 2.6.2. Non-Wearing Time

Even if the subject is active, the measurement result will show inactivity if the accelerometer is not worn. Therefore, the detection of non-wear time is an important issue when measuring PA using an accelerometer. For accelerometers that can be attached directly to the skin of the arm to collect biometric information, such as a SenseWear™ or Actiwatch™, it is possible to detect non-wearing. However, these models are relatively expensive, and as most other accelerometers cannot collect biological information, it is necessary to set detecting conditions for non-wearing.

PA below the detection limit of the accelerometer (e.g., 1.0 METs) also cannot be measured, but most reports refer to non-measurement time as non-wearing time. In such cases, there is a risk of resting behavior being considered non-wearing time. In some studies, a non-measurement time of 60 consecutive minutes has been defined as non-wearing time [59]. Recently, a more precise definition of non-wearing time was used for COPD patients, consisting of 90 consecutive minutes of non-measurement time with an allowance of 2 min of interruption [60,61].

#### 2.6.3. Minimum Required Wearing Time per Day

In previous reports, most studies reported findings without assessing the actual wearing time [62]. Demeyer et al. recommended a wearing time of at least 8 h [63], and the minimum wearing times used were 8 [32,64,65,66], 12 [67], or 20 h [68].

#### 2.6.4. Minimum Number of Valid Days Required

Even after adjusting for environmental and methodological factors, the amount of daily activity can easily change from day to day. Generally, one’s representative PA value is calculated as the average or sum of daily PA values over a certain period of time. Therefore, the minimum number of days required to obtain repeatability should be determined. The repeatability has been evaluated using intraclass correlation coefficients, and the number of days of measurements required in COPD patients has ranged from two to seven [62]. Watz et al. reported that a minimum of two to three days was required in stage IV COPD patients, whereas it was five days in stage I COPD patients [69]. Demeyer et al. recommended measuring for at least four weekdays when assessing step and light activity with a Sensewear Armband™ [63]. After adjusting for environmental and methodological factors, the minimum number of days required to obtain reproducibility was three for both the Actimarker™ [41] and Active Style Pro HJA-750C™ [42].

### 2.7. Patient Conditions Influencing PA

PA in COPD patients can be influenced by several patient factors, including demographic factors, the pulmonary function, dyspnea, exercise capacity, comorbidities, muscular conditions, mental state, and living environment. These factors can lead to inter-patient differences in PA, but the associations are still controversial at present.

The age [5,32,70,71], dyspnea [5,32,70,71,72,73,74], exercise capacity [5,71,72,73], and pulmonary function—including FEV1 [5,32,71,72,75], inspiratory capacity (IC) [70,76], and diffusing capacity [5,73]—are all important factors potentially influencing PA in COPD patients. Depression [75,77], cardiac dysfunction [71], dog walking, grandparenting [78], and employment status [32] can also influence PA in COPD patients.

Muscle quality may also be a relevant factor influencing PA. Muscle mass, especially the cross-sectional area of the erector spine muscle assessed by chest computed tomography (ESM_CSA_) [79], and muscle strength, especially the quadriceps strength [72,80], were shown to be associated with PA in COPD patients. Myokines, especially irisin [81] and growth differentiation factor 11 [82], have been reported to be associated with PA in COPD patients.

Regarding serological tests, C-reactive protein (CRP), fibrinogen, and interleukin-6 values were reported to be associated with PA in COPD patients [78,83,84]. In another report, however, the CRP value was not associated with PA [85]. Furthermore, Taka et al. reported that SIRT1 and FOXO1 mRNA might be associated with PA in COPD patients [86].

### 2.8. Interventions for Improving PA

Evidence concerning the improvement in PA with interventions, including pharmacological management and pulmonary rehabilitation, has been limited, possibly due to a lack of established methodological details, including optimal timing, components, duration, and models for interventions, as well as the evaluation methods. There has also been scant evidence supporting a continued effect over time after the end of intervention [87].

#### 2.8.1. Pharmacological Interventions

Bronchodilator administration has been shown to improve PA in some reports [66,88,89,90,91,92,93,94], albeit depending on the indicator in some cases [95,96,97], while no improvement was seen in other reports [98,99,100,101]. When adjusted for at least two influencing factors, bronchodilators invariably showed beneficial effects on PA with some indicators [66,89,90,91,93,96,97]. However, none of the studies reporting that bronchodilators exerted no beneficial effect on PA performed such adjustments [98,99,100,101]. Consideration of potentially influential factors should therefore be required when evaluating the effects of intervention, and bronchodilators may thus yet be found to improve PA in all COPD patients [18]. Furthermore, when a long-acting muscarinic antagonist (LAMA) and a long-acting beta 2 adrenergic agonist (LABA)/LAMA combination agent were compared in a meta-analysis, PA was found to be significantly improved with LABA/LAMA treatment compared to LAMA [102].

#### 2.8.2. Non-Pharmacological Interventions

Evidence supporting improvements in PA in COPD patients with pulmonary rehabilitation is also limited [87]. However, changes in PA with pulmonary rehabilitation combined with counseling using pedometer feedback have tended to be high [103]. Counseling is predominantly based on the principle of goal-setting and implementation of that goal [104,105]. A positive effect of providing target step count values using an internet-mediated program was seen after 3 or 4 months [106,107] but not after 12 months [108]. The disappearance of this effect after 12 months might have been because even if the patients worked hard to increase the number of steps taken each day, the target value was reviewed and then increased further each week; furthermore, the target value was set according to the current step count without considering the disease condition of each patient. These issues may have made it difficult for patients to remain motivated for a long time. Indeed, half of the participants believed the automated target step counts were too high, and many did not feel comfortable reaching their targets [109].

**Figure 3:**
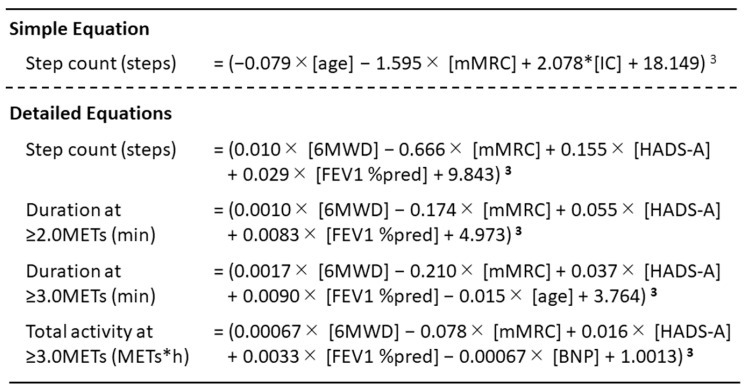
Reference equations of PA using associated factors for COPD patients. PA: physical activity; COPD: chronic obstructive pulmonary disease; mMRC: modified Medical Research Council dyspnea scale; IC: inspiratory capacity; 6MWD: 6-minute walk distance; HADS-A: anxiety score of the Hospital Anxiety and Depression Scale; FEV1%pred: forced expiratory volume in one second percent of predicted value; BNP: serum brain natriuretic peptide. Quoted from references [67,68].

We created referent equations for step count using PA-associated factors for COPD patients (Figure 3) [70,71] and developed a method to set an individual target step count using the current steps and the steps calculated by the equation [110]. Furthermore, a pilot study found that providing a target value was able to increase the step count in patients with innately low step counts [110]. Although an intervention study conducted over a longer duration is required, this target value setting method reflecting the disease condition might be useful for increasing PA in COPD patients.

## 3. The Objective Measurement of SB in COPD

### 3.1. Sedentary Time (ST) in Subjects with Several Conditions

SB is defined as any waking behavior characterized by an energy expenditure ≤1.5 metabolic equivalents (METs) while in a sitting, reclining, or lying-down posture [21,23,24]. ST is one of the frequently used indicators of SB. The concept of ST has been attracting attention in the general population [111,112,113,114,115,116] as well as in patients with cardiovascular disease [117,118,119], diabetes mellitus [118,120,121], and cancer [112,122,123,124,125,126] because of our increasing awareness of our health condition and mortality risk. In COPD patients, ST was reported to be an independent predictor of mortality after adjusting for the duration at ≥3.0 METs and several other variables [25].

### 3.2. Objectively Measured ST and Its Problems

While an accelerometer might detect ST more precisely than a questionnaire, it is difficult to extract the exact ST according to the definition. Investigators should thus treat ST more carefully than PA, as ST accounts for more than half of the total measurement time and does not include the time spent moving during sleep. Associated issues with its measurement can include functional limitations of accelerometers, the exclusion of sleeping time, unification of the total measurement time per day, and minimum required wearing time.

#### 3.2.1. Functional Limitations of Accelerometers

**Figure 4:**
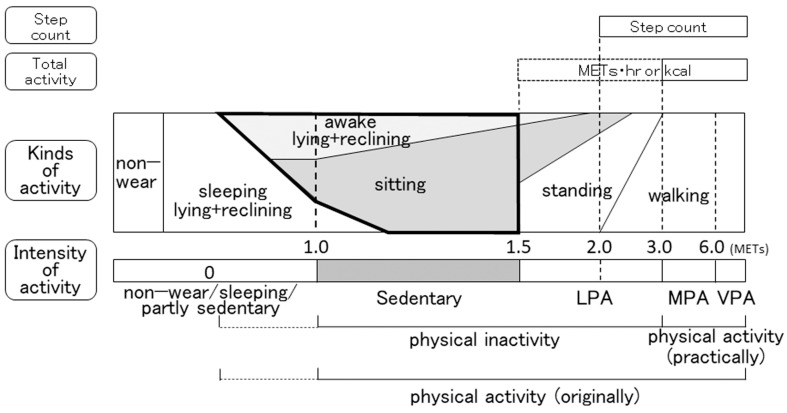
Indicators regarding the physical status based on a comparison of the intensity and kind of activity in cases of COPD. The area surrounded by a bold line indicates sedentary behavior according to the established definition. METs, metabolic equivalents; LPA, light-intensity physical activity; MPA, moderate-intensity physical activity; VPA, vigorous-intensity physical activity.

When an accelerometer that detects the kind of activity is used, the sitting time or sitting + lying-down time is employed as an indicator of ST. In such cases, however, the duration of behavior with an intensity of >1.5 METs while sitting, which is not SB, is included. In our investigation, such instances accounted for 27.5% of the sitting time [127]. Furthermore, the duration spent sleeping while sitting or lying down is also included. These times should be excluded from ST according to the definition (Figure 4) [18]. When an accelerometer that detects the intensity of activity is used, the duration of behavior with an intensity of 1.0–1.5 METs (including both 1.0 and 1.5 METs) [23,66,128] or the ratio of the duration of behavior at 1.0–1.5 METs to the total measurement time may be employed as indicators of ST. In such cases, however, the duration of behavior with an intensity of <1.0 METs is not included in the ST, as it cannot be detected by most accelerometers. Furthermore, the duration spent sleeping while still performing activity with an intensity of 1.0–1.5 METs is also included (Figure 4). These errors are functional limitations of the accelerometer and cannot be avoided. However, while an accurate measurement of ST by definition is difficult regardless of the type of accelerometer used, investigators need to aware that these errors exist and be prepared to compensate for them.

#### 3.2.2. Exclusion of Sleeping Time

Exclusion of sleeping time is another problem for precisely detecting ST. Most behavior during sleep is <1.0 METs in intensity, but during some periods, it can reach ≥1.0 METs, which is incorrectly counted as ST. Furthermore, napping time is difficult to exclude. Since these times cannot be distinguished based on the results obtained with an accelerometer, investigators should keep this error in mind.

#### 3.2.3. Unification of Total Measurement Time per Day

ST can be markedly affected by the total measurement time, as the time spent sitting and lying down accounts for 64% of the total measurement time in COPD patients [4]. Furthermore, subjects tend to feel less of a need to wear accelerometers when they are not active, which might lead to both the total measurement time and ST being shorter than the actual active time. It is therefore best to unify the measurement time if possible.

In some reports, subjects were instructed to wear an accelerometer from the time they woke up to the time they went to bed in order to exclude time spent sleeping [128,129,130]. However, these times varied from day to day and person to person, so the total measurement time varied among measurements. If subjects are instructed to wear an accelerometer at a particular time, such as from 6:00 am to 9:00 pm, some subjects may not have woken up yet at 6:00 am or may have already gone to bed at 9:00 pm. It might therefore be better to ask subjects to wear the accelerometer constantly and extract only the data for a defined period from all of the data obtained [28,66,131]. However, this method has the disadvantage of increasing the burden on the subject due to unnecessary data acquisition during sleep. There is no perfect method for unifying measurement times, so investigators should interpret the data with an understanding of the weaknesses associated with each method.

#### 3.2.4. Minimum Required Wearing Time per Day

Even if the measurement time is unified, wearing time will decrease due to bathing or forgetting to wear the accelerometer. ST can be more strongly influenced by the wearing time than the duration of PA, simply because ST accounts for the majority of the measurement time. The minimum required wearing time was reported to be set to 8 [31], 10 [130,132,133,134], or 12 h [67,128,131,135] in previous studies, and the wearing time tended to be longer for SB assessments than for PA assessments. Further research is needed to confirm the optimal wearing time for evaluating ST.

### 3.3. Lying-Down-Time-to-Sitting-Time Ratio (LSR) in COPD Patients

Both the sitting and lying-down (including reclining) times are included in ST, and both set a lower levels in ST measured in COPD patients compared to those in healthy subjects [4]. However, when the lying-down time was compared with the sitting time, the LSR was larger in COPD patients (23.1%) than in healthy subjects (9.5%) [4]. We investigated the lying-down time and sitting time in COPD patients wearing both intensity-based and activity type-based accelerometers at the same time. The lying-down time accounted for 28.3% of the total wearing time (212 ± 160 min), and the sitting time accounted for 49.4% of the total wearing time (370 ± 123 min), resulting in the LSR being 57.3% [127]. Patients with COPD might spend more time lying down than expected during ST. Furthermore, the duration spent engaged in behaviors at 0 METs, 1.0–1.5 METs, and ≥3.0 METs while sitting accounted for 9.2%, 63.3%, and 27.5% of total time, respectively, and the duration spent engaged in those behaviors while lying down accounted for 29.5%, 62.7%, and 7.8%, respectively [127]. While we have previously described the relationship between the indicators measured with an intensity-based accelerometer and those with an activity type-based accelerometer [18], slight modifications are needed in cases of COPD, as shown in Figure 4.

### 3.4. Interventions for Improving ST

Since no objective measurement method has yet been established, few reports have demonstrated clear intervention effects. We sub-analyzed the results of a crossover study after strictly adjusting for factors affecting the ST and found that the LAMA/LABA combination significantly reduced the ST compared to LAMA alone [66]. This effect was confirmed in a meta-analysis, although the number of reports was only two [102]. Further research with strict adjustment for the influential factors will be required to clarify the effects of interventions.

## 4. Conclusions

The objective measurement of PA and SB is a promising method for clarifying the physical condition of COPD patients; however, several problems remain to be solved. Researchers need to recognize the existence of these problems and the importance of adjusting for them when evaluating.

## Data Availability

Not applicable.
